# Faster healing of radial neck fractures in children treated with the Metaizeau technique compared with percutaneous Kirschner wire fixation: a population-based study

**DOI:** 10.1186/s12891-025-08830-6

**Published:** 2025-07-04

**Authors:** Markus Stöckell, Emmi Lampio, Felicitas Sagstetter, Jaakko Sinikumpu

**Affiliations:** 1 Research Unit of Clinical Medicine, Faculty of Medicine, Oulu Childhood Fracture and Sports Injury Study, University of Oulu, Oulu, Finland; 2 Department of Children and Adolescents, Pediatric Surgery and Orthopaedics, Medical Research Council (MRC), Oulu University Hospital, POB23, Oulu, OYS 90029 Finland; 3 Mannheim Faculty of Medicine, Heidelberg University, Heidelberg, Germany

**Keywords:** Radial neck, Fracture, Children, Surgical fixation, Osteosynthesis, Elastic stable intramedullary nailing, Metaizeau technique, ESIN, Kirschner wires, K-wires

## Abstract

**Background:**

There is increasing experience in the treatment of radial neck fractures using the minimally invasive Metaizeau technique with retrograde elastic stable intramedullary nailing (ESIN) as an alternative to percutaneous Kirschner wire (K-wire) fixation. The main objective of this study was to compare fracture healing between these two techniques.

**Methods:**

This population-based, single-centre study included all 53 consecutive and eligible patients aged < 16 years with radial neck fractures who were operated on at Oulu University Hospital (OUH), Finland, between 2000 and 2020. OUH is the only 24-hour paediatric trauma centre in the area. Radiographs and characteristics of the injury, patient, treatment and radiographic ossification were reviewed, and the treatment groups were compared. Radiographs were taken until bone healing, and all patients were followed up for potential complications for at least two years (mean 89 months, range 33.0 to 170.5, SD 42.7).

**Results:**

All fractures treated with the Metaizeau technique (N = 30) showed complete bone union after 2 months, compared to 18 out of 23 patients treated with K-wire fixation (**P = 0.012**). There was no difference in final bone union between the treatment groups at the final follow-up visit (*P* = 0.178). There was also no difference between the groups in overall clinical recovery (P = 0.431), nor in range of motion outcomes, such as pronation (P = 1.000), supination (P = 0.594) or elbow extension-flexion (P = 0.191).

**Conclusions:**

The Metaizeau technique more frequently resulted in early bone healing than K-wire fixation. Nevertheless, both techniques resulted in good clinical and radiographic outcomes in the long term, making them viable treatment options.

**Clinical trial number:**

Not applicable.

## Introduction

Proximal radius fractures account for 5–10% of elbow fractures [1–3]. They are most common in children aged 7 to 12 years [4]. The most common mechanism of injury is a fall on the outstretched arm with the forearm supinated [4, 5]. There are two main types of proximal radial fracture; metaphyseal fractures of the radial neck, which are more common, and epiphyseal fractures of the radial head [1, 6]. Proximal radial fractures can also occur in association with other elbow injuries, such as elbow dislocation or olecranon fracture [6, 7].

Radial neck fractures are generally not benign, with a reported complication rate of up to 38% [1]. Bone healing of these fractures may be limited due to the fragile vascular supply of the proximal part of the radius [1, 3]. Several factors adversely affect outcomes, including greater fracture displacement, older age and associated injuries [1, 7, 8]. In addition to nonunion, other potential long-term complications include residual valgus deformity and elbow flexion–extension or forearm rotation stiffness [3, 7, 9, 10].

Proximal radial neck fractures can be treated with closed or open reduction with immobilisation or surgical fixation; K-wires and elastic stable intramedullary nails (ESIN) using the Metaizeau technique are available for the osteosynthesis of both radial neck and head fractures [5, 11–13]. Nondisplaced proximal radial neck fractures and displaced fractures with < 30 degrees of angular deformity and < 50% displacement may be treated with non-operative treatment [5, 7, 8, 11]. For other fractures, reduction and surgical fixation are recommended [3, 6, 11].

Because the Metaizeau technique does not disturb the fracture haematoma, it has been suggested that bone healing is improved [3, 8, 10]. In this study, we aimed to compare fracture healing after the Metaizeau technique [3] and K-wire fixation. We also evaluated long-term bone union and clinical recovery.

## Methods

### Study design and materials

All children (< 16 years) who suffered a radial neck fracture in the geographical catchment area of the study centre during the research period 2000–2020, and whose fractures were treated with the Metaizeau technique or K-wire fixation, were included in the study. The International Classification of Diseases (ICD), 10th version, with diagnosis code S52.1, was used for the preliminary identification of the study population. Finally, the diagnoses were confirmed by reviewing the radiographs of all potential patients, resulting in the inclusion of 53 patients. The study centre is the only 24-hour paediatric trauma unit in the region. Patient characteristics, type of injury, treatment and follow-up data, including patient journal notes and radiographs, were collected from the hospital database. Fractures were classified according to the modified Judet classification [3]. Hospital records were reviewed by the first author (SM), who was not involved in the original treatment of the patients.

Satisfactory bridging callus is usually recognised within the first 2 months in these fractures, unless compromised for any reason [11, 14]. Therefore, radiographic bone healing at the 2-month follow-up was taken as the primary outcome. Bone healing was assessed using the principles of the Lane–Sandhu score: fractures were classified as “unioned” if bridging calluses were found in at least 3 cortices out of 4, with or without fracture line consolidation [3].

Any complications, decreased forearm or elbow ROM and overall clinical outcomes were also analysed. In this study, a difference in ROM (supination, pronation, or elbow flexion–extension) of at least 15 degrees compared with the uninjured side was considered an inferior outcome. If the treating surgeon reported limited motion but exact measurements were unavailable, the ROM was considered unsatisfactory. Patients were followed clinically and radiographically until bone healing was achieved. All patients were reviewed for any fracture-related complications treated at the study centre at least two years (mean 89 months, range 33.0 to 170.5, standard deviation (SD) 42.7 months) after surgery.

### Statistics

Categorical variables between groups were analysed using the chi-squared test. The single proportion test was used to assess whether the observed proportion matched the expected population proportion. Normalisation of continuous variables (ROMs) was assessed using the Shapiro–Wilk test. The two independent samples t-test was used for normally distributed continuous variables (extension-flexion), while the Mann–Whitney U-test was used for non-normally distributed variables (pronation and supination). Descriptive statistics such as means, standard deviations (SDs), frequencies (N) and proportions (%) are reported. The threshold for statistically significant difference was set at P < 0.05 (5%) with all values two-tailed. Basic statistics were calculated using SPSS for Windows, version 28.0.1.0 (Armonk, NY, USA, IBM Corp.) and StatsDirect Statistical Software, version 3.3.6 (Wirral, England, StatsDirect Ltd., 2008).

The study was researcher-initiated with no commercial conflict of interest. No patients were contacted for this registry-based study. Therefore, there was no evaluation by the Ethics Committee of the Northern Finland Hospital District, Oulu, Finland. Institutional approval was obtained from the Oulu University Hospital administration before the start of the study.

## Results

### Patient, injury and treatment characteristics

The mean age of the patients was 9.3 years (range 4.0 to 14.0; SD 2.6). Twenty-eight patients had the fracture on the left side (P = 0.784). In total, 24 of the 53 patients were boys (P = 0.497).

The most common type of injury was a fall from a height of ≤ 1 m (N = 35/53), while 18 out of 53 cases involved a fall from a height of more than 1 m. The most common associated recreational activity was trampoline jumping, occurring in 17 out of 53 cases (Table 1).

**Table 1:** Patient and injury characteristics

Mean (range, SD*)	N	%	P-value
Age 9.3 (4 to 14, 2.6)			
Sex			0.292
Male Female	24	45.3	
	29	54.7	
Fracture side			0.784
Right Left	25	47.2	
	28	52.8	
Injury mechanism			0.014
Fall < 1 m Fall > 1 m	35	66.0	
	18	34.0	
Recreational activity			< 0.001
Trampoline jumping	17	32.1	
Playground devices	8	15.1	
Running	7	13.2	
Horseback riding	3	5.7	
Skateboarding	2	3.8	
Skiing	2	3.8	
Other	14	26.4	

* SD = Standard deviation

All fractures were displaced. The most common fracture type was the modified Judet III fracture (N = 35/53), with the remaining fractures classified as Judet IV fractures. There was no difference in fracture type according to the modified Judet classification between the treatment groups (P = 0.912) (Table 2).

**Table 2:** Types of the fractures in the study cases, classified using the modified Judet classification

Judet-classification	All (N = 53) ESIN* (N = 30) K-wire** (N = 23)						P-value
	N	%	N	%	N	%	
III	35	66.0	20	66.7	15	65.2	0.912
IV	18	34.0	10	33.3	8	34.8	

*Elastic stable intramedullary nailing, ***Kirschner wire fixation

### Treatment

**Fig. 1:**
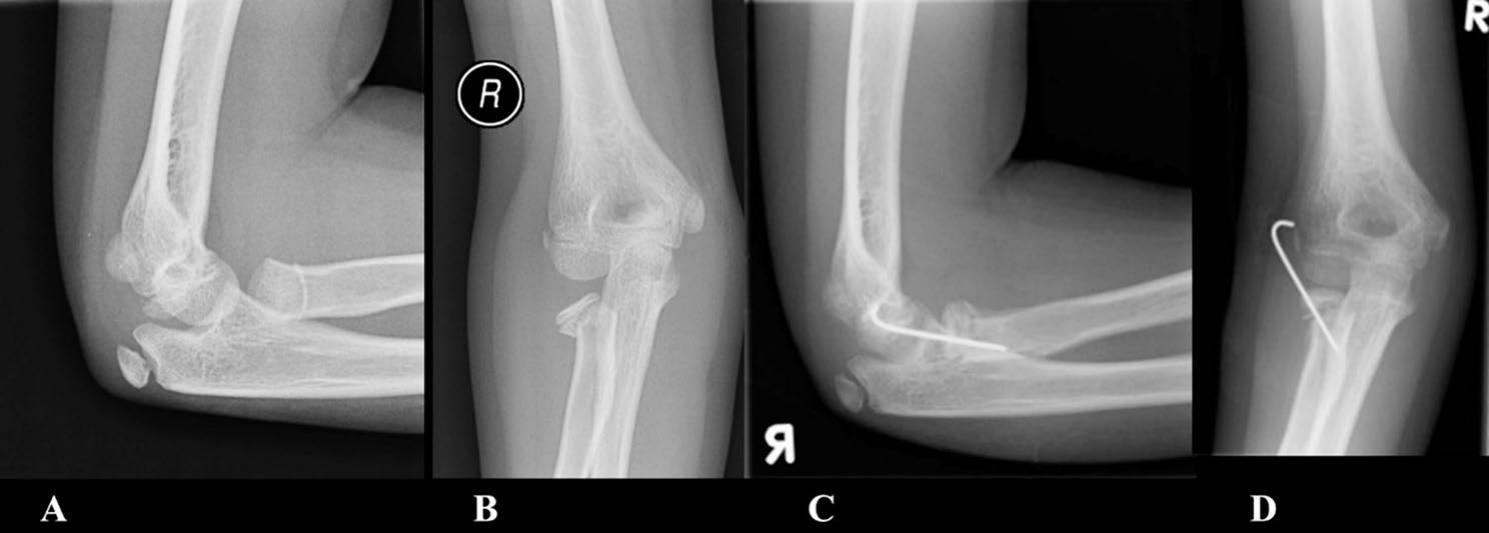
The figures illustares a Judet III radial neck fracture in an 11-year-old, which was surgically treated with a percutaneous K-wire. The fracture united within two months with no postoperative complications, resulting in excellent clinical and radiological outcomes. **A**. Lateral projection, preoperative **B**. Anterior–posterior projection, preoperative **C**. Lateral projection, postoperative **D**. Anterior–posterior projection, postoperative

In total, 30 of 53 patients (57%) were treated with ESIN, while 23 (43%) were treated with K-wires. Half of the patients (N = 28/53) underwent open reduction after a failed closed reduction attempt (Figure 1). Open reduction was performed in 18 out of 35 (51%) Judet III fractures and 10 out of 18 (56%) Judet IV fractures (P = 0.776). Of these, ten out of 18 (56%) and five out of 10 (50%) were in the K-wire group. There was no difference in open vs. closed reduction between the treatment groups (P = 0.114) (Table 3). The Judet classification was not associated with open reduction (P = 0.776).

**Table 3:** Treatment particulars

Reduction	All (N = 53) ESIN* (N = 30) K-wire** (N = 23)						P-value
	N	%	N	%	N	%	
Open	28	56.6	13	43.3	15	65.2	0.114
Closed	25	43.4	17	56.7	8	34.8	

*Elastic stable intramedullary nailing, ***Kirschner wire fixation

### Fracture healing

At 2 months, the majority of fractures (48 out of 53) had stabilised. However, there was a difference between the groups: in the Metaizeau group, all fractures (N = 30) stabilised, whereas in the K-wire group, 5 out of 23 patients had a disturbed union (**P = 0.012**). Three of these 5 cases were initially considered pseudoarthrosis, of which 2 were treated surgically and one eventually healed spontaneously. The other two patients with impaired bone healing had delayed union and slowly stabilised without further intervention. Four (4/5) patients with disturbed bone union were in the open reduction group and two (2/5) were Judet IV fractures. Neither open reduction (P = 0.355) nor differences in Judet classification (P = 1.000) was associated with inferior fracture healing. Finally, complete fracture healing was achieved in all patients at the last follow-up (Table 4).

**Table 4:** Radiological and clinical outcomes of patients with proximal radius fractures treated with elastic stable intramedullary nailing or Kirschner wire fixation

	All N = 53		ESIN*		K-wires**		P-value
	N	%	N	%	N	%	
ROM****							
Pronation	13	24.5	7	53.8	6	46.2	1.000
Supination	15	28.3	9	60.0	6	40.0	0.594
Extension-flexion	16	30.2	10	62.5	6	37.5	0.191
Persistent symptoms							
at 2 months’ mark	12	22.6	7	58.3	5	41.7	0.891
at 6 months’ mark	8	15.1	3	37.5	5	62.5	0.272
at 12 months’ mark	7	13.2	3	42.9	4	57.1	0.431
Complications	14		3	21.4	11	78.6	**0.002**
Neurapraxia	6	42.9	3	50.0	3	50.0	0.729
Disturbed bone healing	5	35.7	0	0	5	100	**0.012**
Delayed union	3	21.4	0	0	3	100	0.076
Nonunion	2	14.3	0	0	2	100	0.184
Surgical site infection	2	21.4	0	0	2	100	0.184
Osteomyelitis	1	7.1	0	0	1	100	0.434

*Elastic stable intramedullary nailing **Kirschner wire fixation

*** Patients for whom range of motion (ROM) data were available

### Functional outcome

There was no difference in clinical recovery, including ROM, between the treatment groups at any time during follow-up (Table 4). The mean pronation movement was 69 degrees in the ESIN group (N = 7, SD 7.2, 95% CI 63.6 to 74.4 degrees) and 66 degrees in the K-wire group (N = 5, SD 9.5, 95% CI 58.4 to 73.6 degrees, P = 1.000). Supination movements were 72 degrees (N = 9, SD 21.6, 95% CI 53.7 to 88.3 degrees) and 71 degrees (N = 6, SD 13.2, 95% CI 62.4 to 79.8 degrees, P = 0.594) in the ESIN and K-wire groups, respectively. Flexion–extension movement was 148 degrees in the ESIN group (N = 10, SD 10.3, 95% CI 141.6 to 154.4 degrees) and 162 degrees in the K-wire group (N = 6, SD 20.6, 95% CI 145.5 to 178.5 degrees, P = 0.191). Three patients from the K-wire group (13%) and 6 patients from the ESIN group (20%), including five patients who had a ROM deficit at the final follow-up, received standardized physical therapy as part of the rehabilitation protocol aimed at achieving full range of motion.

### Complications

**Fig. 2:**
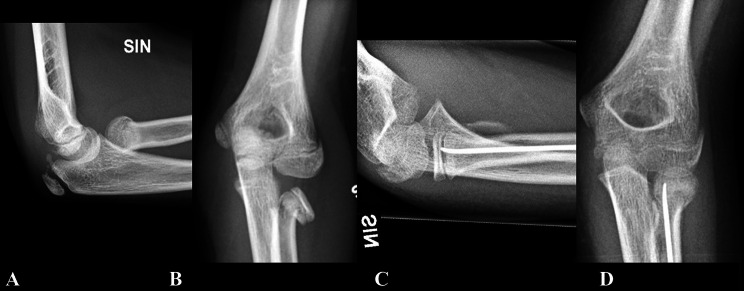
The figures illustrates a Judet IV radial neck fracture in a 9-year-old, treated using the Metaizeau technique. Fracture union was observed at the 2-month follow-up. The patient’s temporary neurapraxia resolved during long-term follow-up, and she achieved excellent clinical and radiological outcomes. **A**. Lateral projection, preoperative **B**. Anterior–posterior, preoperative **C**. Lateral projection, postoperative **D**. Anterior–posterior projection, postoperative

There were 14 complications, of which five involved impaired union and nine were other complications. There were more complications in the K-wire group (N = 11) compared to the ESIN group (N = 3) (**P = 0.002**). Of the 10 patients who experienced complications, 7 were in the open reduction group (P = 0.302). Of these, 4 were in the K-wire group. Temporary neurapraxia occurred in 6 out of 14 cases (Figure 2). Two patients treated with K-wire had surgical site infections. The infection resulted in early removal of the wires; however, another patient progressed to osteomyelitis (Table 4). Overall, 10 different patients experienced complications, with 7 in the K-wire group and 3 in the ESIN group (P = 0.082).

### Clinical recovery

At the 6-month follow-up, 8 out of 53 patients were considered to have unsatisfactory clinical recovery according to the treating surgeon. At the one-year follow-up, 7 out of 53 patients still had not achieved a clinically satisfactory recovery (Table 4). At the final follow-up, five patients had a ROM deficit of 15 degrees or more, due to complications (2 patients with Judet IV fractures, one of them treated with K-wire), associated injuries (2 patients with Judet III fractures, treated with ESIN), or delayed union (one patient with Judet IV fracture, treated with K-wires). Four patients had rotational deficits, three of whom were Judet IV fractures. Two patients had an extension deficit: one with a Judet III fracture and one with a Judet IV fracture, who also had impaired rotational movement. However, the patients achieved the range of motion required for daily activities, and no patient needed any further follow-up or late-stage treatments due to persistent symptoms.

## Discussion

We found that radial neck fractures in children ossified faster when treated with the Metaizeau technique using ESIN compared to K-wire fixation. While all 30 patients in the Metaizeau group stabilised at the early follow-up (2 months), the corresponding number in the K-wire group was 18 out of 23 (**P = 0.012**). This suggests that approximately 1 in 5 patients would have recovered faster if they had been treated with the Metaizeau technique instead of K-wire fixation. This main finding supports the results of a previous study by Du et al. (2019), including 34 radial neck fractures [15], of which 16 were treated with the Metaizeau technique and 18 with K-wire fixation. Du et al. reported that complete fracture healing occurred earlier in the Metaizeau group (mean 5.4 weeks, range 4 − 10 weeks) compared to the K-wire group (mean 6.3 weeks, range 4 − 12 weeks). The slight difference in healing time between the current study and previous studies may be explained by differences in the definition of bone union and follow-up intervals. Nevertheless, 2 months was used in this study to determine bone stabilisation, because radiographically stable ossification of radial neck fractures is usually seen at this time, if not disturbed [11, 14]. In conclusion, faster healing with the Metaizeau technique supports its superiority over K-wire fixation, as bone union is the primary goal in fracture treatment.

There were fewer postoperative complications with the Metaizeau technique, which further supports its superiority over K-wire fixation. The complication rates of 3 patients out of 30 in the Metaizeau group and 7 patients out of 23 in the K-wire group are consistent with findings reported in other studies [10, 16, 17]. Three patients required a second operation due to limited ossification after K-wire fixation. Previous literature reports varying results regarding complications: Kalem et al. (2018) studied 21 radial neck fractures [18], 10 of which were treated with the Metaizeau technique and 11 with K-wires, finding no statistically significant difference in complication rates. Tarallo et al. investigated 20 patients: 12 of whom were treated with percutaneous pinning and 8 with ESIN [5]. They found more complications in the K-wire group and concluded that ESIN was preferable. The sample sizes of both studies were smaller than in the present study, which makes the current study important in strengthening the existing literature.

Despite the varying time to bone stability, all fractures eventually healed completely, and long-term fracture healing did not differ between the treatment groups. This is an encouraging finding, though not surprising, given that childhood fractures typically heal well [19]. Good long-term bone union after both procedures has also been reported in previous studies [1, 5, 10, 20]. Therefore, in the authors’ opinion, both ESIN and K-wire fixation remain viable surgical procedures for the treatment of radial neck fractures, with the choice depending on the surgeon’s skills and experience.

The Metaizeau technique for displaced radial neck fractures in children is a minimally invasive procedure that may result in less soft tissue and epiphyseal damage [6, 10, 13–15]. The entry point is distant from the fracture, which avoids compromising the fracture haematoma. Additionally, it is technically a relatively simple procedure if patient selection is appropriate. In contrast, blind K-wire insertion may require multiple attempts before satisfactory fixation is achieved [2, 6, 21]. This can further compromise the fragile circulation of the radial head. Furthermore, the wires usually approach the bone through the haematoma, which is detrimental to callus formation. Additionally, the posterior interosseous nerve (PIN) may be injured in percutaneous K-wire fixation, leading to transient or permanent nerve palsy [12, 22]. Transient neurological deficits following proximal radius injuries or interventions have been reported in 1.3–2.0% of cases [2, 17]. In this study, 2 patients treated with K-wires suffered from transient PIN damage, accounting for 3.8% of the patients.

The majority of the patients (N = 48/53) achieved excellent or good long-term clinical outcomes. These predominantly good long-term results are consistent with previous literature [2, 4, 10, 11, 14, 23], despite the poor reputation of proximal radius injuries. Although most studies comparing these two surgical techniques have concluded that the Metaizeau technique is superior, there are also conflicting results [6, 21, 24]. Tian et al. studied 62 children with radial neck fractures [12], of whom 37 were treated with K-wires and 25 with the Metaizeau technique. They reported 4 clinical complications in the ESIN group and one in the K-wire group (P = 0.640). The time to union was shorter in the K-wire group (5.05 ± 0.58 weeks) than in the ESIN group (5.96 ± 1.33 weeks) (P = 0.001). However, the mean age of the patients in that study (7.5 years) was lower than in this study patients and in radial neck fracture patients in general. This age difference limits direct comparison. In younger children, the smaller diameter of the ESIN that can be used due to a smaller intramedullary space may compromise reduction. Satisfactory rotational stiffness of the implant is essential for correctly repositioning the radial head, and thinner ESIN may not provide the necessary support for this.

In this study, 17 out of 53 patients had associated injuries, which is consistent with the literature [25, 26]. Fractures with associated injuries increase the complexity of management and may partly explain the generally poorer outcomes of radial neck fractures [1, 7]. Many studies have reported that open reduction results in inferior outcomes and is associated with a higher incidence of complications compared to closed reduction and minimally invasive fixation [1, 7, 11, 12, 20, 21]. Avascular necrosis can result from disruption of the blood supply, either due to the injury itself or as a consequence of surgical manipulation during open reduction [23]. Judet type IV fractures of the proximal radius are more likely to require open reduction [27]. Higher rates of avascular necrosis and pseudoarthrosis have been reported following open reduction in cases of Judet type IV fractures [2, 28–30]. Moreover, worse outcomes have been noted for Judet IV fractures compared to JudetIII fractures [31, 32]. However, in this study, neither open reduction nor Judet classification was associated with impaired bone healing or a higher incidence of adverse events.

Similar to other studies, girls predominated, accounting for 29 out of the 53 participants [6, 10, 25]. One explanatory factor may be the cause of the fracture: trampoline jumping, the most common cause (17/53), and this activity is particularly popular among girls [33]. Trampoline jumping has remained popular throughout the 2000s, suggesting that the incidence of these high-energy fractures is unlikely to decrease in the near future [33, 34]. Additionally, the majority (70%) of all fractures in children involve the upper extremities, and their incidence has continued to increase [34, 35].

This study has several strengths. It included all patients with radial neck fractures treated at Oulu University Hospital District over a 20-year period. There were no other paediatric 24-hour trauma centres in the area, so the enrolment was considered comprehensive. While there may have been isolated cases treated at other institutions in the area or elsewhere during their travel, this is unlikely to have significantly impacted the results. The long study period ensured an adequate sufficient sample size, despite the generally low annual prevalence of proximal radial fractures. Radial neck fractures are not among the most common fractures in children and adolescents. The mean age of the patients in this study (9 years) is consistent with previous studies [5, 11, 25]. As this was a public hospital, each patient received the same treatment regardless of economic situation or insurance status. Treatment decisions were made individually by the treating surgeons for each case. The surgeons were all consultants, with few operations performed by a registrar under the supervision of an experienced consultant. Patient follow-up was comprehensive, and details of clinical and radiographic follow-up data were widely available. Bone healing was assessed using predetermined, clear criteria.

There are some limitations. The range of motion of the elbow and forearm was not available for all patients. The primary outcome, time to bone union, was not available as a continuous variable because patients followed a standard follow-up protocol with clinical and radiographic investigations conducted as part of routine care. Therefore, a comparison of proportion (united fractures per all fractures) was performed between the two treatment groups at each follow-up visit. Due to the retrospective nature of the study, each patient’s surgical method was chosen based on the surgeon’s preference and patients were not randomised into groups. However, there were no differences between the groups in essential particulars, such as classification or the need for open reduction. While the sample size was relatively large for this rare fracture, it remained too small to allow for reliable subgroup analyses, such as evaluating the impact of the reduction method combined with the surgical technique on bone healing. Larger prospective and randomised studies are needed to confirm these findings and facilitate more comprehensive subgroup analyses.

## Conclusion

Radial neck fractures in children treated with either the Metaizeau technique or K-wires ultimately achieve good radiological results. However, the Metaizeau technique more frequently resulted in early healing compared to K-wire fixation. There were also fewer complications in the Metaizeau group. These findings support the use of the Metaizeau technique as the primary method for treating radial neck fractures in children.

## Data Availability

The datasets generated and analyzed during the current study are not publicly available due to privacy or ethical restrictions but are available from the corresponding author upon reasonable request.

## Abbreviations

ESIN: Elastic Stable Intramedullary Nail/Nailing
ICD: International Classification of Diseases
K-wire: Kirschner wire
ROM: Range Of Motion
SD: Standard Deviation
